# Inflammatory Markers Mediate the Association Between Cardiovascular Health and Chronic Inflammatory Airway Diseases: A Cross-Sectional Study on the Population Aged 50 Years and Above From NHANES 2013–2018

**DOI:** 10.1155/mi/2128440

**Published:** 2025-06-16

**Authors:** Zhaoqi Yan, Xiufan Du, Jing Zhang

**Affiliations:** ^1^Cardiovascular Department, Guang'anmen Hospital, China Academy of Chinese Medical Sciences, Beijing, China; ^2^Department of Rehabilitation Medicine, The Third Hospital of Nanchang, Nanchang People's Hospital, Nanchang, Jiangxi, China; ^3^The Second Department of Infectious Disease, Shanghai Fifth People's Hospital, Fudan University, Shanghai, China; ^4^Center of Community-Based Health Research, Fudan University, Shanghai, China

**Keywords:** cardiovascular health, chronic inflammatory airway diseases, inflammatory marker, Life's Essential 8, National Health and Nutrition Examination Survey

## Abstract

**Objective:** This cross-sectional study collected data from participants in the National Health and Nutrition Examination Survey (NHANES) from 2013 to 2018, aiming to investigate the association between cardiovascular health (CVH) and chronic inflammatory airway diseases (CIADs).

**Methods:** Single-factor and multiple-factor logistic regression analyses were used to explore the relationship between CVH levels assessed based on Life's Essential 8 (LE8) scores and CIAD. Subgroup and interaction analyses were conducted and restricted cubic splines (RCSs) were used to evaluate the linear or nonlinear relationship between LE8 scores and CIAD. Mediation analysis was conducted to investigate the mediating role of inflammatory markers between CVH levels and CIAD.

**Results:** A total of 5736 participants were included in this study. When participants with high CVH levels (LE8 scores: 80–100) were used as the reference standard, the average risk of CIAD increased by 1.78 times in participants with moderate CVH (odds ratio (OR) = 1.78, 95% confidence interval (CI) (1.25, 2.51)) and the risk of CIAD in participants with low CVH increased by 2.45 times (OR = 2.45, 95% CI (1.53, 3.94)). Subgroup analysis indicates that older age and comorbidities are significantly associated with CIAD and there is no interaction between various covariates and CVH levels. RCS showed a negative linear relationship between LE8 scores and CIAD. Mediation analysis results revealed that neutrophil (NEU) count and Systemic Immune-Inflammation (SII) Index significantly mediated the relationship between CVH and CIAD.

**Conclusion:** Moderate and low levels of CVH pose a threat for developing CIAD, with inflammatory factors playing a major mediating role in this association.

## 1. Introduction

Chronic inflammatory airway diseases (CIADs) are a group of long-term respiratory conditions caused by infections or allergic reactions, including chronic obstructive pulmonary disease (COPD), asthma, and chronic bronchitis [1]. These diseases share common characteristics such as chronic airway inflammation leading to airway remodeling, airflow limitation, and decreased lung function. In severe cases, CIADs may also lead to complications such as respiratory failure and heart disease, significantly impacting patients' health and quality of life. According to data from the World Health Organization (WHO), CIADs have become one of the most common chronic diseases globally, with their incidence and mortality rates expected to continue rising in the coming decades [2, 3]. Chronic respiratory diseases, along with cardiovascular disease (CVD) and cancer, are among the top three causes of disease-related mortality, accounting for approximately 7.0% of all-cause mortality [1]. As the primary classification of chronic respiratory diseases, CIAD should not be underestimated. CIAD and CVD often coexist and have poor prognoses, with pathophysiological connections between the two including pulmonary hyperinflation and systemic inflammation [4, 5]. Both conditions share common risk factors such as aging, smoking, unhealthy diet, and sedentary lifestyle [6, 7]. In fact, comorbid CVD significantly impacts the incidence, progression, and mortality of respiratory disorders. Studies demonstrate that 30%–50% of COPD-related deaths are attributable to cardiovascular complications [8], while mitigating pulmonary/systemic inflammation has proven effective in improving survival and quality of life in these patients [9]. A meta-analysis revealed a 25% increased risk of major adverse cardiovascular events (MACEs) in COPD patients after adjusting for conventional cardiovascular risk factors compared to non-COPD populations [10]. Population-based research indicates that the actual cardiovascular risk in COPD patients exceeds predictions from traditional risk assessment tools like QRISK3 by 52% [11]. Recent National Health and Nutrition Examination Survey (NHANES) findings further emphasize the necessity of systematic cardiovascular screening for individuals with COPD and chronic bronchitis [12]. Notably, asthma may independently contribute to MACE development [13], with emerging evidence elucidating bidirectional pathophysiological relationships between these conditions [14]. These findings collectively highlight that cardiopulmonary interactions may extend beyond traditional risk factor paradigms. Therefore, maintaining cardiovascular health (CVH) should constitute an essential component in the clinical management of CIAD.

In 2022, the American Heart Association (AHA) introduced a revised ideal CVH metric based on the Life's Simple 7 (LS7) launched in 2010, referred to as Life's Essential 8 (LE8). This new metric incorporates sleep-related scoring criteria and updates some of the LS7 scoring parameters [15]. LE8 is a comprehensive scoring system that is more sensitive to interindividual and intraindividual differences, relating to various physiological and pathological responses such as aging, oxidative stress, and inflammation levels [16–18]. The updated LE8 consists of four health behaviors (sleep, smoking, physical activity, and diet) and four health factors (obesity, lipids, blood glucose, and blood pressure).

The association of LE8 with other non-CVDs is gradually being explored [19, 20]. Previous studies have indicated that higher LS7 levels are associated with normal lung function and a reduced risk of COPD [21]. However, the relationship between LE8 and CIAD remains elusive. Considering the close connection between pulmonary diseases and CVD [6], promoting LE8-related CVH measurement standards is likely to be a preventive and management strategy to alleviate the burden of CIAD.

This cross-sectional study is based on data from the NHANES in the United States, investigating the relationship between CVH levels, as measured by LE8 scores, and CIAD risk among participants aged 50 years and above. Additionally, it explores whether inflammatory markers play an intermediary role between these two aspects.

## 2. Materials and Methods

### 2.1. Study Population in NHANES

In this study, we specifically analyzed data collected from 2013 to 2018, excluding samples based on the following criteria: (1) missing parameters related to the assessment of the LE8 scores; (2) missing data from the CIAD questionnaire; (3) age under 50; (4) missing covariate datasets.

### 2.2. Exposure Variables—CVH Evaluation

In this study, according to AHA guidelines, we collected data on eight factors: diet, physical activity, nicotine exposure, sleep health, body mass index (BMI), lipids, blood glucose, and blood pressure to calculate the LE8 scores, which serves as a standard for evaluating CVH levels. The LE8 scores is out of 100, with higher scores indicating better CVH. Dietary information was obtained through self-reported food frequency questionnaires, which included dietary quality assessment methods proposed by the AHA. Physical activity was assessed based on the number of minutes of moderate or vigorous physical activity per week, while nicotine exposure information included smoking history. Sleep health was evaluated based on average sleep duration per night, and BMI was calculated from measured weight and height. Lipid and blood glucose levels were assessed based on corresponding physiological indicators, while blood pressure was classified according to measurements of systolic and diastolic pressure. (For detailed calculation process of LE8 scores and relevant explanations from the AHA Association, please refer to Table S1.) The scores for these eight CVH indicators range from 0 to 100, with the total LE8 score calculated as the unweighted average. According to AHA recommendations, we categorized CVH into high CVH level (80–100), moderate CVH level (50–79), and low CVH level (0–49) [15, 22].

### 2.3. Outcome Variables—CIAD

The definition of CIAD is based on three chronic respiratory diseases included in the NHANES questionnaire: COPD, asthma, and chronic bronchitis. The questionnaire asked: “Has a doctor or other health professional ever told you that you have or had chronic bronchitis (MCQ160K), asthma (MCQ010), or COPD (MCQ160O)?” Participants who answered “yes” to any CIAD-related question were defined as having the disease.

### 2.4. Inflammation-Related Indicators

Blood samples were analyzed for complete blood counts using the Beckman Coulter DxH 800 instrument at the NHANES mobile examination center (MEC), providing blood cell distribution for all participants. We included several inflammation-related laboratory tests and calculated inflammation indicators based on these tests: white blood cell (WBC) count, neutrophil (NEU) count, NEU-to-lymphocyte ratio (NLR) [23], and Systemic Immune-Inflammation (SII) Index [24]. The units for lymphocyte, NEU, and platelet counts are 1000 cells/µL, and SII levels are calculated as platelet count × NEU count/lymphocyte count.

### 2.5. Covariates

Covariate information for this study was collected from NHANES demographic data, questionnaires, and laboratory tests. We categorized the covariates into three main groups: baseline demographic data (age, gender, and race), lifestyle factors (marital status, education level, BMI, serum cotinine level, and smoking status), and comorbidities (hypertension, hyperlipidemia, diabetes, and CVD) [25]. Specific definitions are provided in Table S2.

### 2.6. Statistical Analysis

A complex sampling design was implemented to ensure nationally representative estimates, with all analyses adjusted for survey design and weighted variables. The sample weight used was “WTMEC2YR" and new sample weights were calculated by dividing the original 2-year sample weights by 3. Strata (stratification) and PSU (primary sampling unit) are assessed through SDMVPSU and SDMVSTRA. Continuous variables are presented as mean ± standard deviation (SD), while categorical variables are expressed as individual counts (*N*) and percentages (%). Weighted *t*-tests (for continuous variables) or weighted chi-square tests (for categorical variables) were used to assess differences between CIAD and non-CIAD participants. For the classification of CVH, the same approach was employed to evaluate differences among the three groups.

In evaluating the association between CVH and CIAD, we first fitted a crude multivariable logistic regression model, followed by stepwise adjustments for covariates. Model 1 adjusted for baseline demographic data, Model 2 further adjusted for lifestyle factors on top of Model 1, and Model 3 adjusted for comorbidities based on Model 2. In Model 3, a multivariable logistic regression model was used to assess the significance of the interaction between CVH and covariates on CIAD, along with conducting subgroup analyses. For sensitivity analysis, we employed multiple imputation to address missing covariates and validate the robustness of the logistic regression model. The methodology comprised the following steps: Using the random forest algorithm within the R “mice” package, we performed multiple imputation with a default of five iterations. Data imputation quality was subsequently assessed through convergence diagnostic plots and the baseline characteristics of the study cohort were presented postimputation.

Additionally, in the restricted cubic splines (RCSs) model, we treated the LE8 scores as a continuous variable, employing three knots at the 10^th^, 50^th^, and 90^th^ percentiles to explore whether the association between LE8 scores and CIAD risk was linear or nonlinear [26].

Mediation analysis was conducted using the “Mediation” package in R software to explore whether inflammatory factors had a potential mediating effect on the association between CVH (exposure) and CIAD (outcome). The total effect, direct effect, and mediation effect were analyzed based on three pathways (a, b, and c) using multivariable logistic regression in Model 3: (1) The total effect (Path a) assessed the relationship between CVH levels and CIAD. (2) The direct effect (Path b) evaluated the relationship between CVH levels and inflammatory markers (mediators). The mediation effect analysis (Path c) measured the impact of inflammatory markers on the relationship between CVH levels and CIAD. The effect of inflammatory markers on the link between CVH levels and CIAD was evaluated through the mediation effect (Path c). The proportion of the mediation effect was calculated as: (total effect−direct effect) × 100/total effect. The *p*-values less than 0.05 for all statistical analyses in this study were considered significant and all results are presented as odds ratios (OR) with corresponding 95% confidence intervals (95% CIs). The statistical analysis was conducted using R-4.2.2.

## 3. Results

### 3.1. The Baseline Characteristics of the Participants With CIAD and Non-CIAD

**Figure 1:**
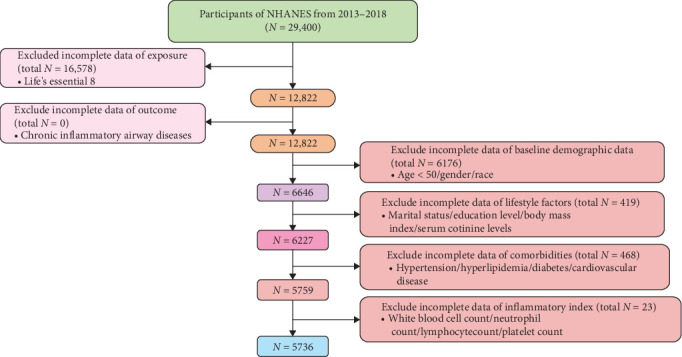
Flowchart of the study design and participants excluded from the study.

In this study, a total of 5736 participants were included, with 1244 participants being CIAD (Figure 1). In terms of the basic characteristics of the patients, CIAD patients were generally older than the non-CIAD population. They had a higher proportion of males, participants with a moderate level of education, those who were overweight or obese, smokers, and participants with CVD. However, there was no significant difference in terms of ethnicity or marital status between the two groups (Table 1). It is worth noting that the LE8 scores of CIAD patients were, on average, six points lower than those of the non-CIAD population. The proportion of participants with a low level of CVH was significantly higher among CIAD patients. Various inflammatory markers such as WBC, NEU, NLR, and SII showed varying degrees of elevation in CIAD patients, indicating that they were facing a significant cardiovascular risk crisis.

Furthermore, we observed that the proportion of participants at low CVH level, whether in the CIAD population or its subgroups (COPD, chronic bronchitis, and asthma), was increasing. Additionally, the levels of inflammatory markers were also on the rise in these participants.

Following random forest imputation, the imputed data for all variables demonstrated distributional consistency with original observations (Figure S1), confirming that the randomness of the data was maintained through the interpolation process. The postimputation cohort retained 6646 participants, with baseline characteristics showing strong concordance with the original dataset (Table S3). It also shows that the CVH level is worthy of attention.

In the flowchart, the green box represents the total number of initial participants from three NHANES cycles (2013–2018). The pink box on the left quantifies data missingness for the exposure variable (CVH status defined by LE8) and the outcome variable (CIAD). The orange-red boxes on the right hierarchically display missing data distributions for baseline demographic characteristics, lifestyle parameters, comorbidities, and inflammatory biomarkers from top to bottom. Rectangular annotations along the central axis indicate the progressive sample size reduction following exclusion criteria application, ultimately defining the analytical cohort.

### 3.2. Associations Between CVH and CIAD

By constructing multiple linear regression models, we found a significant association between the level of CVH and the risk of CIAD when using high CVH level (LE8 scores: 80–100) as the reference standard. Particularly in Model 3, adjusted for baseline demographics, lifestyle factors, and comorbidities, moderate and low CVH levels were still significantly positively correlated with the risk of CIAD. Compared to participants with high CVH level, the average risk of CIAD increased by 1.78 times in participants with moderate CVH level (OR = 1.78, 95% CI (1.25, 2.51)) and it reached 2.45 times in participants with low CVH level (OR = 2.45, 95% CI (1.53, 3.94)). Furthermore, this risk decreased with an increase in LE8 scores (OR = 0.99, 95% CI (0.98, 1.00); Table 2). The sensitivity analysis showed that the data after interpolation showed a high degree of consistency (Table S4), which supported the robustness of the results.

Further subgrouping CIAD, similar results were observed for asthma patients. The average risk of asthma increased by 1.58 times (OR = 1.58, 95% CI (1.09, 2.29)) and 2.29 times (OR = 2.29, 95% CI (1.33, 3.95)) in participants with moderate and low CVH level, respectively. For COPD, after adjusting for various covariates in Model 3, low CVH level was significantly positively associated with the risk of COPD (OR = 2.77, 95% CI (1.03, 7.40)). Chronic bronchitis only showed an association with CVH level in the crude model (unadjusted for covariates) and Model 1 (adjusted for baseline demographic data). Moreover, the increase in SII among inflammatory markers is most significantly associated with an increased risk of CIAD (Table S5).

**Figure 2:**
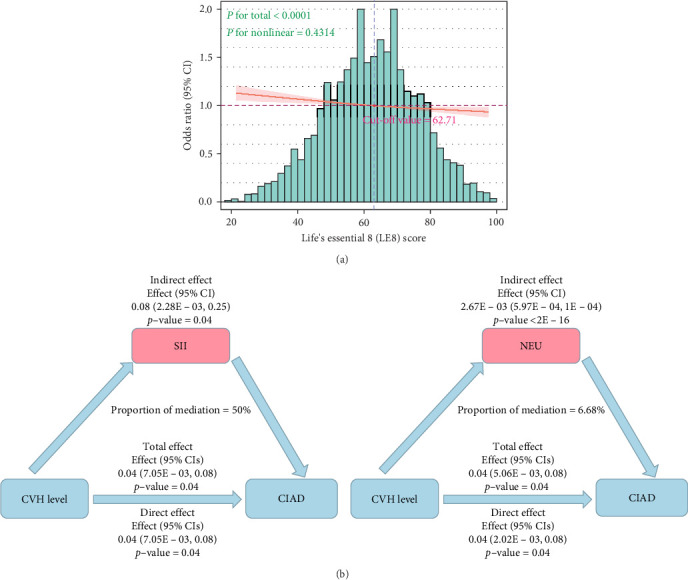
Restricted cubic spline (RCS) model and mediation effects. (A) The RCS analysis demonstrates the multivariate adjusted odds ratio (OR) of CIAD across LE8 scores (*x*-axis: LE8; *y*-axis: OR). The green histogram reflects the frequency distribution of participants' LE8 scores, with its approximately normal distribution supporting sample representativeness. The red solid line represents Model 3 (adjusted for baseline demographics, lifestyle factors, and comorbidities), with light-red shaded areas indicating 95% confidence intervals from RCS regression. The intersection of the purple dashed line and the red curve (LE8 = 62.71) defines the risk threshold, below which CIAD risk escalates significantly. (B) Mediation analysis of inflammatory biomarkers in the relationship between CVH and CIAD. The left panel illustrates the mediating role of the Systemic Immune-Inflammatory (SII) Index, while the right panel demonstrates the mediation by neutrophil (NEU) count. Pink-bordered areas represent the mediated effects, with blue and green text indicating the total effect and direct effect values, respectively. Results are presented as effect sizes, 95% confidence intervals, and *p*-values.

Using the RCS model to further fit the relationship between LE8 scores and CIAD, in Model 3 adjusted for covariates, we found a linear relationship between the two without any nonlinear correlation (*p*=0.43, Figure 2A), which is consistent with the trend test results in the logistic regression. The LE8 scores cut-off point where the risk of CIAD significantly increased was determined to be 62.71.

### 3.3. Subgroup Analysis of Covariates and Mediation Effects of Inflammatory Markers

We conducted a subgroup analysis on items that showed statistical differences between the CIAD and non-CIAD populations as presented in Table 1. In the age group of 50–60 years, only low CVH level had a risk impact on CIAD. However, as age increased, the risk of CIAD also began to emerge in the moderate CVH level group, with the OR showing a significant increase. All gender subgroups exhibited notable differences, demonstrating the applicability of this indicator across diverse populations. Additionally, participants with certain comorbidities (hypertension, hyperlipidemia, CVD, and diabetes) showed an increased risk of CIAD.

It is noteworthy that there were no interactions observed in any of the subgroup analyses, indicating that these subgroup analyses do not have a decisive impact on the findings of this study (Table 3). Furthermore, mediation analysis revealed that inflammatory markers play a mediating role in the relationship between CVH levels and CIAD. Specifically, NEU and SII significantly mediated the correlation between CVH levels and CIAD, with NEU and SII explaining 6.68% and 50% of the association, respectively. This highlights that inflammatory factors indeed constitute a substantial portion of the relationship between CVH levels and the risk of CIAD (Figure 2B, Tables S5 and S6).

## 4. Discussion

This study represents the first large-scale cross-sectional research aimed at elucidating the relationship between CVH and CIAD based on inflammatory markers. Our findings indicate that moderate and low CVH levels correlate with an elevated risk of CIAD. The RCS analysis demonstrated a linear relationship between CVH levels and CIAD risk, suggesting that maintaining a LE8 scores above 62.71 is advisable for the American population to mitigate the risk of CIAD. Moreover, two inflammatory markers, NEU and SII, were found to mediate the association between CVH and CIAD. This mediation effect underscores the significant role that inflammation plays in the relationship between CVH and the risk of developing CIAD. These findings provide strong support for promoting LE8–based CVH level initiatives as a preventive strategy against CIAD. By emphasizing the importance of maintaining optimal CVH, we can potentially reduce the incidence of CIAD and improve overall public health outcomes.

Previous studies assessing CIAD risk predominantly relied on single-system tools (e.g., St. George's Respiratory Questionnaire [27] and Veterans SF-12 [28]). Most of these studies focused mainly on the comorbidity and mortality of CVD and CIAD [29], neglecting the impacts of health behaviors and cardiovascular-specific metrics. The various components of LE8 and their relationship with CIAD have been extensively studied, such as lack of exercise, overweight and obesity, smoking, poor diet, sleep disorders, hypertension, dyslipidemia, and dysglycemia. Cardiovascular events are also considered common comorbidities of various CIAD [4, 30, 31], all of which suggest that maintaining a good level of CVH is a powerful means to reduce the occurrence of CIAD. In contrast, our study introduces three distinct advantages using LE8: (1) By systematically integrating multidimensional risk factors, LE8 not only enhances the mechanistic understanding of CIAD risk but also methodologically extends prior research paradigms. This framework, grounded in robust evidence-based foundations. (2) LE8′s continuous scoring system enables precise CVH quantification for early CIAD prevention, surpassing binary CVD diagnosis. (3) We pioneer the exploration of inflammatory mediation pathways (e.g., systemic immune-inflammatory index and NEU count) to elucidate LE8-CIAD mechanisms, a gap in prior research. Studies have indicated that excessive energy intake, consumption of “fast food,” and a lack of fruits, vegetables, and whole grains are dietary-related risk factors for CIAD [32, 33], Therefore, it is necessary to use HEI-2015 as a guideline for a healthy diet. Additionally, maintaining healthy and regular sleep patterns [34, 35] and reducing nicotine exposure are protective factors against diseases related to aging, particularly for CIAD [36, 37]. A previous NHANES study also suggested that obesity may be a risk factor for triggering CIAD and increase the associated mortality risk [38]. The relationship between blood pressure, lipid levels, and CIAD remains unclear; however, some studies have shown that systolic blood pressure (SBP) and COPD are associated with a higher risk of CVD [39]. Participants with COPD experience increased CVD risk at higher SBP levels [40]. Both obesity and high lipid levels are associated with increased oxidative stress in the body and airways, potentially due to an imbalance of adipokines and reduced antioxidant defenses, leading to damage in alveolar epithelial cells [41]. Moreover, considering the intricate relationships among sleep, diet, obesity, and physiological markers like blood pressure and lipid levels, amalgamating these factors could serve as a more effective assessment approach. Moreover, CIAD is associated with aging, one of the characteristics of which is systemic chronic inflammation, a hallmark pathological change in the development of CIAD. Multiple unhealthy categories within the LE8 indicator have also been repeatedly shown to disrupt immune homeostasis and induce and exacerbate inflammatory processes. Last, the health behaviors included in LE8 are associated with a reduced risk of mood disorders [42, 43] and mood disorders have also been identified as risk factors for CIAD [44, 45], with a pathophysiological relationship existing between dyspnea, hyperventilation, and psychological disorders. Anxiety can also serve as a triggering factor for starting smoking [46]. These unhealthy factors can activate pro-inflammatory pathways, thereby inducing CIAD [32, 47].

Our research has identified that the threat of developing CIAD exists at moderate and low CVH levels. Additionally, the RCS model revealed a linear negative correlation between LE8 scores and CIAD. We were able to precisely quantify that when the LE8 scores reaches 62.71, the risk of CIAD significantly increases. In our study, nearly 70% of the participants were at a moderate level of CVH, indicating a significant risk of CIAD among the American population.

Our research also indicates that inflammatory factors partially explain the association between CVH and CIAD, a relationship that has not been explored before. Inflammatory markers tend to increase as LE8 scores decrease and inflammation levels are significantly higher in the CIAD population. Atherosclerosis, as a precursor to CVD and similar to CIAD, is a systemic inflammatory disease [48]. Therefore, the decreased CVH levels represented by a decrease in LE8 scores also reflect systemic inflammation. SII and NEU play crucial roles in the association between CVH and CIAD, with SII explaining up to 50% of the efficacy. Previous studies have suggested that assessing SII levels can be part of screening, diagnosing, and managing lung function impairment and coronary artery disease (CAD) patients [49, 50] and a recent meta-analysis has highlighted the prognostic value of SII for CAD [51]. Due to SII being a novel systemic inflammation assessment marker, it better reflects the overall immune and inflammatory status of the body. In fact, inflammatory responses not only lead to repeated structural damage and repair in the lungs but also activate adaptive immune responses, aiding in the repair and barrier function of damaged lung tissue. Therefore, maintaining good lifestyle habits is crucial in balancing inflammation, immunity, tissue damage, and repair. NEU is the most predominant WBC in the blood and plays a crucial role in the nonspecific immune system. Currently, observational and genetic studies have confirmed a positive association between NEU count and CVD [52]. The airway damage closely associated with NEU and CIAD is evident, with NEU having additional adverse effects on the airways, including airway remodeling leading to narrowing and increased mucus secretion [53], increased smooth muscle reactivity [54], and decreased lung function [55].

In conclusion, various adverse lifestyle factors and complications related to CVH contribute to sustained local and systemic inflammation in the body, which is a significant factor in triggering CIAD.

However, this study still has several limitations that merit attention: (1) The cross-sectional design cannot establish causal relationships between CVH levels and CIAD, merely reflecting their correlation. Although we meticulously adjusted for various covariates and explored the impacts of inflammatory factors, these adjustments remain insufficiently comprehensive. To verify the causal mechanisms and investigate long-term effects, further randomized controlled trials or longitudinal cohort studies are necessary to confirm our findings. (2) This study exclusively enrolled participants aged 50 years and above. This decision was based on the observation that individuals over 50 represent not only a high-risk group for CIAD [56] but also exhibit elevated cardiovascular comorbidity risks (Table S7). Furthermore, we found that CIAD definitions in participants under 50 were predominantly influenced by asthma (Figure S2), and the disproportionately high prevalence of asthma contradicted the study's primary focus on CIAD analysis. Refined age stratification demonstrated that participants aged 50 and above formed a more representative cohort for CIAD investigation (Figure S3). Nevertheless, future validation of our findings in other databases, such as the China Health and Retirement Longitudinal Study (CHARLS), and expansion of population coverage to broaden the clinical applicability of results remain crucial directions for further exploration.

## 5. Conclusion

Our research has revealed that there is a threat of developing CIAD at moderate and low CVH levels, with inflammatory factors playing a crucial mediating role in this process. Our findings suggest that the American population should gradually improve their health behaviors and lifestyle factors to reduce the risk of CIAD by lowering systemic inflammation levels.

## Figures and Tables

**Table 1:** Characteristics of participants by CIAD or non-CIAD.

Characteristic	Overall, *N* = 5736 (100%)^a,b^	Non-CIAD, *N* = 4492 (78%)^a,b^	CIAD, *N* = 1244 (22%)^a,b^	*p* Value^c^
Age (years)*⁣*^*∗*^	63.5 (9.0)	63.3 (9.1)	64.1 (8.6)	0.024
Gender*⁣*^*∗∗*^	—	—	—	0.003
Female	2985 (54%)	2264 (52%)	721 (60%)	—
Male	2751 (46%)	2228 (48%)	523 (40%)	—
Race	—	—	—	0.066
Non-Hispanic White	2473 (75%)	1845 (74%)	628 (77%)	—
Non-Hispanic Black	1261 (9.3%)	983 (9.2%)	278 (9.9%)	—
Mexican American	734 (4.9%)	624 (5.3%)	110 (3.4%)	—
Other Race—including multi-racial	652 (6.5%)	546 (7.0%)	106 (5.8%)	—
Other Hispanic	616 (4.4%)	494 (4.5%)	122 (3.9%)	—
BMI (Kg/m^2^)*⁣*^*∗∗∗*^	—	—	—	<0.001
Normal (≥18.5, <25)	1297 (23%)	1056 (24%)	241 (20%)	—
Overweight (≥25, <30)	2002 (34%)	1642 (36%)	360 (28%)	—
Obese (≥30)	2437 (43%)	1794 (40%)	643 (51%)	—
Marital	—	—	—	0.073
Divorced	3253 (63%)	2616 (64%)	637 (59%)	—
Married	2288 (35%)	1729 (34%)	559 (39%)	—
Never married	195 (2.1%)	147 (2.1%)	48 (2.1%)	—
Serum cotinine*⁣*^*∗∗∗*^	46 (122)	39 (115)	72 (142)	<0.001
Smoking status*⁣*^*∗∗∗*^	—	—	—	<0.001
Current	885 (14%)	601 (12%)	284 (22%)	—
Former	1888 (34%)	1431 (32%)	457 (38%)	—
Never	2963 (52%)	2460 (56%)	503 (40%)	—
Education*⁣*^*∗∗∗*^	—	—	—	<0.001
High educational level	1400 (32%)	1167 (34%)	233 (24%)	—
Medium educational level	3109 (56%)	2376 (54%)	733 (63%)	—
Primary educational level	1227 (12%)	949 (12%)	278 (14%)	—
Cardiovascular disease*⁣*^*∗∗∗*^	1108 (16%)	388 (29%)	720 (13%)	<0.001
Heart failure	333 (4.2%)	155 (10%)	178 (2.6%)	—
Coronary heart disease	462 (7.6%)	171 (15%)	291 (5.7%)	—
Angina pectoris	252 (4.2%)	105 (9.8%)	147 (2.7%)	—
Heart attack	453 (6.5%)	159 (12%)	294 (5.0%)	—
Stroke	380 (5.0%)	118 (8.3%)	262 (4.2%)	—
Hypertension*⁣*^*∗∗∗*^	—	—	—	<0.001
Hypertension	3750 (59%)	886 (66%)	2864 (58%)	—
Nonhypertension	1986 (41%)	358 (34%)	1628 (42%)	—
Hyperlipidemia	—	—	—	0.2
Hyperlipidemia	4705 (82%)	1041 (84%)	3664 (82%)	—
Nonhyperlipidemia	1031 (18%)	203 (16%)	828 (18%)	—
Diabetes*⁣*^*∗∗∗*^	—	—	—	<0.001
Diabetes	1879 (26%)	469 (32%)	1410 (25%)	—
Nondiabetes	3857 (74%)	775 (68%)	3,082 (75%)	—
CVH level *⁣*^*∗∗∗*^	—	—	—	<0.001
Low-level (0–49)	1012 (14%)	680 (12%)	332 (23%)	—
Moderate-level (50–79)	4048 (69%)	3219 (70%)	829 (69%)	—
High-level (80–100)	676 (17%)	593 (19%)	83 (8.1%)	—
LE8 scores*⁣*^*∗∗∗*^	65 (14)	66 (14)	60 (15)	<0.001
WBC*⁣*^*∗∗∗*^	7.22 (3.82)	7.12 (4.14)	7.60 (2.25)	<0.001
NEU*⁣*^*∗∗∗*^	4.26 (1.60)	4.18 (1.57)	4.54 (1.68)	<0.001
NLR*⁣*^*∗*^	2.31 (1.25)	2.27 (1.18)	2.42 (1.47)	0.044
SII*⁣*^*∗∗*^	526 (316)	512 (290)	573 (393)	0.004

*Note:* NHANES 2013–2018, *N* = 5736.

Abbreviations: BMI, body mass index; CIAD, chronic inflammatory airway diseases; CVH, cardiovascular health; LE8, Life's Essential 8; NEU, neutrophil count; NHANES, National Health and Nutrition Examination Survey; NLR, neutrophil-to-lymphocyte ratio; SII, Systemic Immune-Inflammation Index; WBC, white blood cell count.

^a^Mean ± SD for continuous; n (%) for categorical.

^b^ *t*-test adapted to complex survey samples; chi-squared test with Rao and Scott's second-order correction.

^c^ *⁣* ^*∗*^ *p*  < 0.05; *⁣*^*∗∗*^*p*  < 0.01; *⁣*^*∗∗∗*^*p*  < 0.001.

**Table 2:** Weighted multivariable adjusted logistic regression analysis of the risk of CIAD associated with different levels of CVH.

Regression model	Crude model OR (95% CI)	Model 1 OR (95% CI)	Model 2 OR (95% CI)	Model 3 OR (95% CI)
CIAD				
High-level (80–100)	Reference	Reference	Reference	Reference
Moderate-level (50–79)	2.32 (1.73, 3.11)*⁣*^*∗∗∗*^	2.37 (1.76, 3.20)*⁣*^*∗∗∗*^	1.77 (1.26, 2.48)*⁣*^*∗∗*^	1.78 (1.25, 2.51)*⁣*^*∗∗*^
Low-level (0–49)	4.55 (3.29, 6.31)*⁣*^*∗∗∗*^	4.71 (3.36, 6.60)*⁣*^*∗∗∗*^	2.64 (1.71, 4.07)*⁣*^*∗∗∗*^	2.45 (1.53, 3.94)*⁣*^*∗∗∗*^
*P* for trend	0.01	9.31E−03	8.21E−03	1.50E−03
LE8	0.98 (0.97, 0.99)*⁣*^*∗∗∗*^	0.97 (0.97, 0.98)*⁣*^*∗∗∗*^	0.97 (0.97, 0.98)*⁣*^*∗∗∗*^	0.99 (0.98, 1.00)*⁣*^*∗∗*^
COPD				
High-level (80–100)	Reference	Reference	Reference	Reference
Moderate-level (50–79)	4.15 (1.94, 8.88)*⁣*^*∗∗∗*^	4.19 (1.94, 9.03)*⁣*^*∗∗∗*^	2.03 (0.87, 4.77)	1.89 (0.77, 4.64)
Low-level (0–49)	11.10 (5.05, 24.2)*⁣*^*∗∗∗*^	12.00 (5.37, 26.8)*⁣*^*∗∗∗*^	3.46 (1.38, 8.68)*⁣*^*∗*^	2.77 (1.03, 7.40)*⁣*^*∗*^
*P* for trend	0.02	0.02	0.02	0.01
LE8	0.98 (0.97, 0.99)*⁣*^*∗∗∗*^	0.96 (0.95, 0.97)*⁣*^*∗∗∗*^	0.98 (0.97, 1.00)*⁣*^*∗∗*^	0.99 (0.97, 1.01)
Asthma				
High-level (80–100)	Reference	Reference	Reference	Reference
Moderate-level (50–79)	1.72 (1.24, 2.39)*⁣*^*∗∗*^	1.78 (1.27, 2.49)*⁣*^*∗∗∗*^	1.64 (1.14, 2.36)*⁣*^*∗*^	1.58 (1.09, 2.29)*⁣*^*∗*^
Low-level (0–49)	2.90 (1.98, 4.24)*⁣*^*∗∗∗*^	2.96 (1.99, 4.40)*⁣*^*∗∗∗*^	2.47 (1.48, 4.12)*⁣*^*∗∗∗*^	2.29 (1.33, 3.95)*⁣*^*∗∗*^
*P* for trend	8.90E−03	6.60E−03	5.90E−03	5.08E−03
LE8	0.98(0.97, 0.99)*⁣*^*∗∗∗*^	0.98 (0.97, 0.99)*⁣*^*∗∗∗*^	0.98 (0.97, 0.99)*⁣*^*∗∗∗*^	0.99 (0.98, 1.00)*⁣*^*∗∗*^
Chronic bronchitis				
High-level (80–100)	Reference	Reference	Reference	Reference
Moderate-level (50–79)	3.08 (1.65, 5.75)*⁣*^*∗∗∗*^	3.17 (1.67, 6.03)*⁣*^*∗∗∗*^	1.85 (0.92, 3.71)	1.83 (0.88, 3.80)
Low-level (0–49)	5.54 (2.90, 10.6)*⁣*^*∗∗∗*^	5.77 (2.99, 11.1)*⁣*^*∗∗∗*^	2.14 (0.99, 4.61)	1.88 (0.80, 4.41)
*P* for trend	1.20E−03	4.14E−04	7.17E−04	3.27E−03
LE8	0.98 (0.97, 0.99)*⁣*^*∗∗∗*^	0.97 (0.96, 0.97)*⁣*^*∗∗∗*^	0.98 (0.97, 0.99)*⁣*^*∗*^	0.99 (0.97, 1.00)

*Note:* Multiple logistic regression model: Model 1: adjusted for baseline demographic data; Model 2: adjusted for baseline demographic data and lifestyle factors; Model 3: adjusted for baseline demographic data, lifestyle factors, and comorbidities.

Abbreviations: CIAD, chronic inflammatory airway diseases; COPD, chronic obstructive pulmonary disease; CVH, Cardiovascular health.

*⁣* ^*∗*^ *p*  < 0.05.

*⁣* ^*∗∗*^ *p*  < 0.01.

*⁣* ^*∗∗∗*^ *p*  < 0.001.

**Table 3:** Subgroup analysis of the correlation between CVH and CIAD.

Subgroup	Moderate CVH levels (50–79) OR (95% CI)	Low CVH levels (0–49) OR (95% CI)	Interaction *p* Value
Age	—	—	*p*=0.24
50–60	1.38 (0.81, 2.37)	2.00 (1.01, 4.02)*⁣*^*∗*^	—
61–75	2.04 (1.15, 3.62)*⁣*^*∗*^	2.87 (1.34, 6.12)*⁣*^*∗∗∗*^	—
>75	4.03 (1.47, 11.10)*⁣*^*∗∗*^	4.70 (1.52, 14.5)*⁣*^*∗∗*^	—
Sex	—	—	*p*=0.99
Female	1.75 (1.03, 2.98)*⁣*^*∗*^	2.36 (1.22, 4.59)*⁣*^*∗∗*^	—
Male	1.74 (1.05, 2.90)*⁣*^*∗*^	2.43 (1.22, 4.82)*⁣*^*∗*^	—
Education	—	—	*p*=0.14
Primary educational level	3.10 (1.78, 5.41)*⁣*^*∗∗∗*^	6.47 (2.57, 16.3)*⁣*^*∗∗∗*^	—
Medium educational level	1.10 (0.65, 1.84)	1.35 (0.72, 2.56)	—
High educational level	3.00 (1.09, 8.26)*⁣*^*∗*^	3.94 (1.16, 13.4)*⁣*^*∗*^	—
BMI (Kg/m^2^)	—	—	*p*=0.55
Normal (<25)	2.09 (0.65, 6.76)	2.99 (0.80, 11.1)	—
Obese (≥30)	1.9 (1.18, 3.06)*⁣*^*∗*^	1.85 (0.79, 4.32)	—
Overweight (≥25, <30)	1.71 (0.82, 3.58)	2.81 (1.26, 6.24)*⁣*^*∗*^	—
Smoking status	—	—	*p*=0.11
Current	4.25 (0.45, 40.5)	7.25 (0.64, 81.9)	—
Former	1.8 (0.90, 3.58)	2.72 (1.23, 6.05)*⁣*^*∗*^	—
Never	1.89 (1.13, 3.19)*⁣*^*∗*^	1.79 (0.86, 3.73)	—
Hypertension	—	—	*p*=0.09
Yes	2.02 (1.06, 3.83)*⁣*^*∗*^	2.56 (1.24, 5.30)*⁣*^*∗*^	—
No	1.42 (0.85, 2.37)	2.96 (1.44, 6.07)*⁣*^*∗∗*^	—
CVD	—	—	*p*=0.06
Yes	1.57 (1.06, 2.31)*⁣*^*∗*^	2.21 (1.28, 3.79)*⁣*^*∗∗*^	—
No	5.15 (1.91, 13.9)*⁣*^*∗∗*^	6.44 (2.53, 16.4)*⁣*^*∗∗∗*^	—
Diabetes	—	—	*p*=0.65
Yes	1.9 (1.31, 2.76)*⁣*^*∗∗*^	2.75 (1.54, 4.91)*⁣*^*∗∗∗*^	—
No	0.83 (0.19, 3.55)	1.03 (0.23, 4.70)	—

*Note:* Subgroup analysis adjustment factors: baseline demographic data, lifestyle factors, and comorbidities, excluding subgroup variables, and the reference object in the subgroup is high level of CVH (Life's Essential 8 scores: 80–100).

Abbreviations: CIAD, chronic inflammatory airway diseases; CVH, cardiovascular health.

*⁣* ^*∗*^ *p*  < 0.05.

*⁣* ^*∗∗*^ *p*  < 0.01.

*⁣* ^*∗∗∗*^ *p*  < 0.001.

## Data Availability

The data supporting the study findings are available from the corresponding authors upon reasonable request.

## Supporting Information

Supporting Information Table S1. Definition and scoring approach for the American Heart Association's Life's Essential 8 score. Table S2. Definition of covariates. Figure S1. Data imputation diagnostic plot after handling missing covariates using random forest method. Table S3. Characteristics of participants by CIAD or non-CIAD after filling in covariate missing values (NHANES 2013−2018, *N* = 6646). Table S4. Weighted multivariate adjusted logistic regression analysis of CIAD risk associated with different CVH levels after filling in covariate missing values. Table S5. Weighted multivariable adjusted logistic regression analysis of the risk of CIAD associated with different inflammatory markers. Table S6. Mediating Role of Inflammatory Indicators in the Relationship between CVH Levels and CIAD. Table S7. Characteristics of participants by age (NHANES 2013−2018, *N* = 5736). Figure S2. Trend line chart of CIAD and its subtypes with age cutoff at 50 years. Figure S3. Trend line chart of CIAD and its subtypes across different age groups.
