# Supplementary material for: Inflammatory Markers Mediate the Association Between Cardiovascular Health and Chronic Inflammatory Airway Diseases: A Cross-Sectional Study on the Population Aged 50 Years and Above From NHANES 2013–2018
Source: Mediators Inflamm. 2025 Jun 16;2025:2128440. doi: 10.1155/mi/2128440 (PMC12185202; doi:10.1155/mi/2128440)
Supplement: Supporting Information — Table S1. Definition and scoring approach for the American Heart Association's Life's Essential 8 score. Table S2. Definition of covariates. Figure S1. Data imputation diagnostic plot after handling missing covariates using random forest method. Table S3. Characteristics of participants by CIAD or non-CIAD after filling in covariate missing values (NHANES 2013−2018, N = 6646). Table S4. Weighted multivariate adjusted logistic regression analysis of CIAD risk associated with different CVH levels after filling in covariate missing values. Table S5. Weighted multivariable adjusted logistic regression analysis of the risk of CIAD associated with different inflammatory markers. Table S6. Mediating Role of Inflammatory Indicators in the Relationship between CVH Levels and CIAD. Table S7. Characteristics of participants by age (NHANES 2013−2018, N = 5736). Figure S2. Trend line chart of CIAD and its subtypes with age cutoff at 50 years. Figure S3. Trend line chart of CIAD and its subtypes across different age groups. [file 2128440.f1.docx]

Inflammatory markers mediate the association between cardiovascular health and Chronic Inflammatory Airway Diseases: A cross-sectional study on the population aged 50 and above from NHANES 2013-2018

[Table S1. Definition and scoring approach for the American Heart Association’s Life’s Essential 8 score 2](#_Toc190019141)

[Table S2. Definition of covariates 7](#_Toc190019142)

[Figure S1. Data imputation diagnostic plot after handling missing covariates using random forest method 8](#_Toc190019143)

[Table S3. Characteristics of participants by CIAD or Non-CIAD after filling in covariate missing values. (NHANES 2013-2018, N = 6,646) 10](#_Toc190019144)

[Table S4. Weighted multivariate adjusted logistic regression analysis of CIAD risk associated with different CVH levels after filling in covariate missing values. 12](#_Toc190019145)

[Table S5. Weighted multivariable adjusted logistic regression analysis of the risk of CIAD associated with different inflammatory markers. 14](#_Toc190019146)

[Table S6. Mediating Role of Inflammatory Indicators in the Relationship between CVH Levels and CIAD. 15](#_Toc190019147)

[Table S7. Characteristics of participants by age. (NHANES 2013-2018, N = 5,736) 16](#_Toc190019148)

[Figure S2. Trend line chart of CIAD and its subtypes with age cutoff at 50 years 19](#_Toc190019149)

[Figure S3. Trend line chart of CIAD and its subtypes across different age groups 21](#_Toc190019150)

# Table S1. Definition and scoring approach for the American Heart Association’s Life’s Essential 8 score

| Domain | CVH Metric | Measurement | Quantification and Scoring of CVH Metric |
| --- | --- | --- | --- |
| **Health Behaviors** | Diet | Healthy Eating Index-2015 diet score percentile | Quantiles of DASH-style diet adherence  **Scoring (Population):**  Points Quantile  100 ≥95^th^ percentile (top/ideal diet)  80 75^th^ – 94^th^ percentile  50 50^th^ – 74^th^ percentile  25 25^th^ – 49^th^ percentile  0 1^st^ – 24^th^ percentile (bottom/least ideal quartile) |
|  | Physical activity | Self-reported minutes of moderate or vigorous physical activity per week | **Metric:** Minutes of moderate (or greater) intensity activity per week  **Scoring:**  Points Minutes  100 ≥150  90 120 – 149  80 90 – 119  60 60 – 89  40 30 – 59  20 1 – 29  0 0 |
|  | Nicotine exposure | Self-reported use of cigarettes or inhaled nicotine- delivery system | **Metric:** Combustible tobacco use and/or inhaled NDS use; or secondhand smoke exposure  **Scoring:**  Points Status  100 Never smoker  75 Former smoker, quit ≥5 yrs  50 Former smoker, quit 1 - <5 yrs  25 Former smoker, quit <1 year, or currently using inhaled NDS  0 Current smoker  Subtract 20 points (unless score is 0) for living with active indoor smoker in home |
|  | Sleep health | Self-reported average hours of sleep per night | **Metric:** Average hours of sleep per night  **Scoring:**  Points Level  100 7 – <9  90 9 – <10  70 6 – <7  40 5 – <6 or ≥10  20 4 – <5  0 <4 |
| **Health Factors** | Body mass index | Body weight (kg) divided by height squared (m^2^) | **Metric:** Body mass index (kg/m^2^)  **Scoring:** Points Level  100 <25  70 25.0 – 29.9  30 30.0 – 34.9  15 35.0 – 39.9  0 ≥40.0 |
|  | Blood lipids | Plasma total and HDL-cholesterol with calculation of non-HDL-cholesterol | **Metric:** Non-HDL-cholesterol (mg/dL)  **Scoring:**  Points Level  100 <130  60 130 – 159  40 160 – 189  20 190 – 219  0 ≥220  If drug-treated level, subtract 20 points |
|  | Blood glucose | Fasting blood glucose or casual hemoglobin A1c | **Metric:** Fasting blood glucose (mg/dL) or Hemoglobin A1c (%)  **Scoring:**  Points Level  100 No history of diabetes and FBG <100 (or HbA1c < 5.7)  60 No diabetes and FBG 100 – 125 (or HbA1c 5.7-6.4) (Pre-diabetes)  40 Diabetes with HbA1c <7.0  30 Diabetes with HbA1c 7.0 – 7.9  20 Diabetes with HbA1c 8.0 – 8.9  10 Diabetes with Hb A1c 9.0 – 9.9  0 Diabetes with HbA1c ≥10.0 |
|  | Blood pressure | Appropriately measured systolic and diastolic blood pressure | **Metric:** Systolic and diastolic blood pressure (mm Hg)  **Scoring:**  Points Level  100 <120/<80 (Optimal)  75 120-129/<80 (Elevated)  50 130-139 or 80-89 (Stage I HTN)  25 140-159 or 90-99  0 ≥160 or ≥100  Subtract 20 points if treated level |

**This table is from the review published by the American Heart Association (AHA) in 2022:** Lloyd-Jones DM, Allen NB, Anderson CAM, Black T, Brewer LC, Foraker RE, Grandner MA, Lavretsky H, Perak AM, Sharma G, Rosamond W; American Heart Association. Life's Essential 8: Updating and Enhancing the American Heart Association's Construct of Cardiovascular Health: A Presidential Advisory From the American Heart Association. Circulation. 2022 Aug 2;146(5):e18-e43.

**Instructions:**

**Diet**: Dietary information was obtained using self-reported food frequency questionnaires. Participants with dietary information for 2 days were included in the data analysis (participants with data for only 1 day were excluded). The American Heart Association (AHA) has proposed a new method for assessing diet quality based on population levels (percentile values corresponding to 0, 25, 50, 80, and 100 points for the 1st-24th, 25th-49th, 50th-74th, 75th-94th, and 95th percentile, respectively).

**Physical Activity**: Physical activity was assessed based on self-reported minutes of moderate or vigorous physical activity collected through questionnaires on a weekly basis. Physical activity time was categorized into weekly intervals of 0, 1-29, 30-59, 60-89, 90-119, 120-149, and ≥150 minutes, corresponding to 0, 20, 40, 60, 80, 90, and 100 points.

**Nicotine Exposure**: Nicotine exposure information was collected through self-reported data in the questionnaire. In addition to combustible cigarettes, the AHA has included the use of other nicotine delivery systems such as electronic cigarette devices, e-cigarettes, and secondhand tobacco smoke in the definition of Life's Essential 8. Nicotine exposure is categorized into five groups, including current smokers, former smokers (<1 year quit), current users of inhaled nicotine delivery systems, former smokers (quit 1 to <5 years), former smokers (quit ≥5 years), and never smokers, corresponding to 0, 25, 50, 80, and 100 points. For adults with active indoor smokers at home, deduct 20 points.

**Sleep Health**: Sleep health is a new indicator in the Life's Essential 8 goals, based on self-reported average nightly sleep time collected through questionnaires. Nightly sleep hours are categorized into <4, 4-<5 hours, 5-<6 hours or ≥10 hours, 6-<7 hours, 9-<10 hours, 7-<9 hours, corresponding to 0, 20, 40, 70, 90, and 100 points.

**Body Mass Index**: Objective measurements of weight and height are used to calculate BMI (i.e., weight in kilograms divided by height in meters squared). BMI levels are categorized as ≥40.0, 35.0-39.9, 30.0-34.9, 25.0-29.9, and <25.0 kg/m², corresponding to 0, 15, 30, 70, and 100 points.

**Lipids**: Blood samples are used to measure total cholesterol and high-density lipoprotein (HDL) cholesterol. Non-HDL cholesterol is calculated as total cholesterol minus HDL cholesterol. Non-HDL cholesterol levels are categorized as ≥220, 190-219, 160-189, 130-159, and <130 mg/dL, corresponding to 0, 20, 40, 60, and 100 points.

**Blood Glucose**: Fasting blood samples are used to measure fasting blood glucose (FBG). Both fasting and non-fasting blood samples are used to measure HbA1c. Blood glucose levels are categorized as diabetes, HbA1c ≥ 10.0%, diabetes HbA1c 9.0-9.9%, diabetes HbA1c 8.0-8.9%, diabetes HbA1c 7.0-7.9%, diabetes HbA1c < 7.0%, non-diabetes and FBG 100-125 mg/dL or HbA1c 5.7-6.4%, no history of diabetes and FBG < 100 mg/dL or HbA1c < 5.7%, corresponding to 0, 10, 20, 30, 40, 60, and 100 points.

**Blood Pressure**: Blood pressure is measured using an appropriately sized cuff. Blood pressure levels are categorized as systolic pressure ≥ 160 mmHg or diastolic pressure ≥ 100 mmHg, 140-159 or 90-99 mmHg, 130-139 or 80-89 mmHg, 120-129/<80 mmHg, and <120/80 mmHg, corresponding to 0, 25, 50, 75, and 100 points. Subtract 20 points if blood pressure levels are under treatment.

# Table S2. Definition of covariates

| **Covariates** | **Definition** |
| --- | --- |
| Race | The original racial classifications from NHANES were condensed into five categories: Mexican American, Non-Hispanic White, Non-Hispanic Black, Other Race - Including Multi-Racial, and Other Hispanic. |
| Marital status | Marital status was categorized as divorced, married, or unmarried. |
| Education level | Education level was classified as Primary Educational (less than 9th grade, 9-11th grade), Medium Educational Level (High Educational Level and High School Graduate/GED or Equivalent), and High Educational Level (College Graduate or above and Some College or AA degree). |
| BMI | BMI classifications were defined as normal (<25 kg/m²), obese (≥30 kg/m²), and overweight (≥25 kg/m² but <30 kg/m²). |
| Smoking status | Smokers (defined as having smoked more than 100 cigarettes in their lifetime) and Non-Smokers (defined as having smoked less than 100 cigarettes in their lifetime). |
| Hypertension | Hypertension is defined as systolic blood pressure ≥130 mmHg or diastolic blood pressure ≥80 mmHg, with self-reported diagnosis or use of antihypertensive medication. |
| Hyperlipidemia | Hyperlipidemia was defined by any of the following criteria: total cholesterol levels equal to or exceeding 200 mg/dL, triglyceride levels equal to or exceeding 150 mg/dL, male HDL-C levels below 40 mg/dL, female HDL-C levels below 50 mg/dL, or low-density lipoprotein cholesterol (LDL-C) levels equal to or exceeding 130 mg/dL. |
| Diabetes | The standards for diabetes are as follows: (1) self-reported diabetes diagnosis; (2) use of diabetes medication; (3) HbA1c > 6.5%; (4) fasting blood glucose ≥ 7 mmol/L. |
| Cardiovascular disease | Cardiovascular disease was defined as a positive response to any of the following questions: “Has a doctor or other health professional ever told you that you have congestive heart failure (CHF), coronary heart disease (CHD), angina, a heart attack, or a stroke?” |

# Figure S1. Data imputation diagnostic plot after handling missing covariates using random forest method


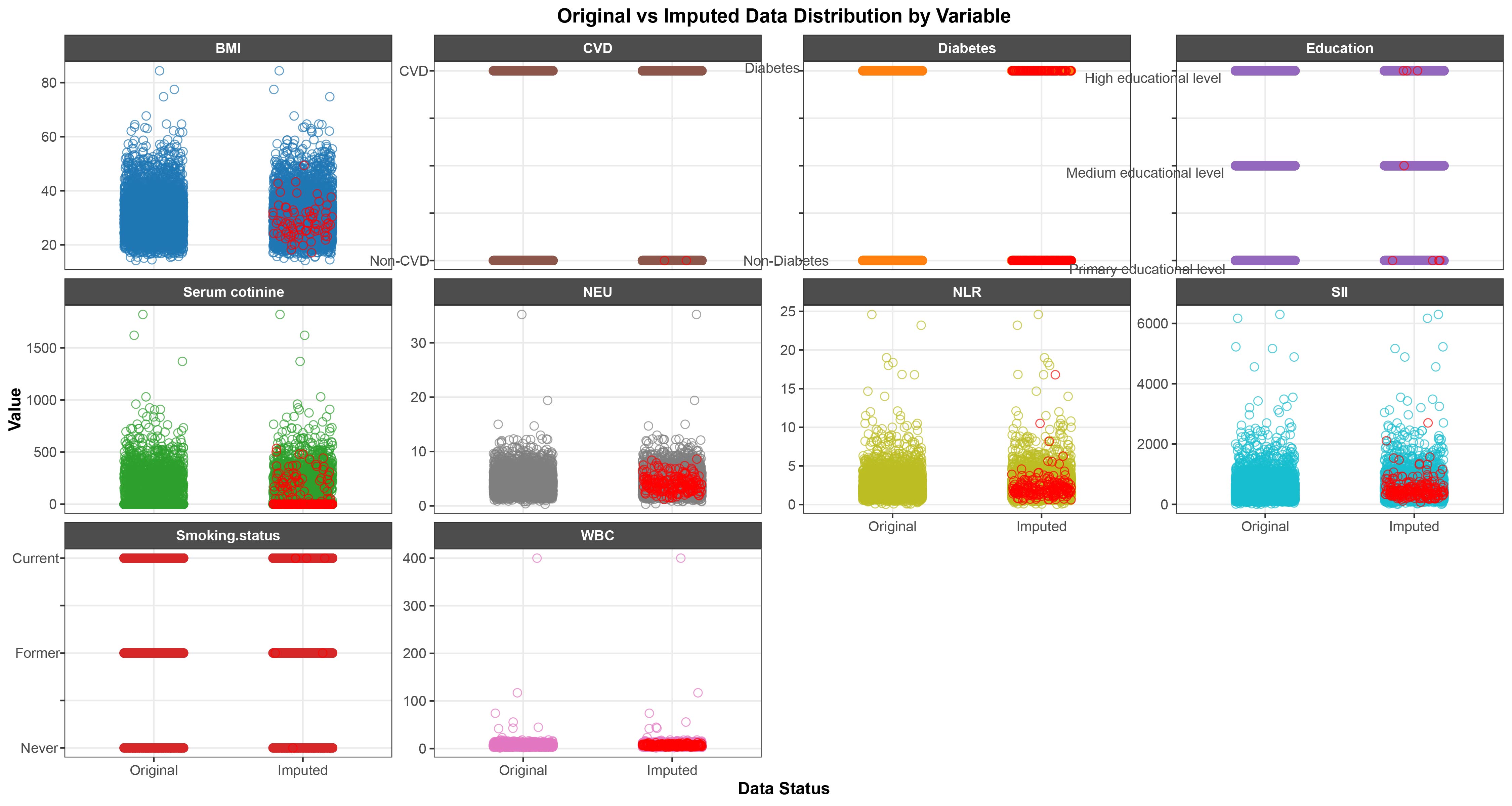


There are 10 covariate indicators with missing values, and the missing data were imputed using the Random Forest (RF) method from the "mice" package. The left side of each plot shows the original data bubble plot, while the right side displays the bubble plot of the data after RF imputation, with the red areas on the right representing the imputed data. It can be intuitively observed that the distribution of the imputed data for each variable is generally consistent with that of the original data, indicating that the imputed data is reasonable.

# Table S3. Characteristics of participants by CIAD or Non-CIAD after filling in covariate missing values (NHANES 2013-2018, N = 6,646)

| **Characteristic** | **Overall**, N = 6,646 (100%)^1,2^ | **Non-CIAD,** N = 5,203 (78%)^1,2^ | **CIAD**, N = 1,443 (22%)^1,2^ | **P Value**^3^ |
| --- | --- | --- | --- | --- |
| **Age (years) *** | 63.6 (9.0) | 63.5 (9.1) | 64.2 (8.7) | **0.049** |
| **Gender***** |  |  |  | **<0.001** |
| *Female* | 3,428 (54%) | 2,599 (52%) | 829 (60%) |  |
| *Male* | 3,218 (46%) | 2,604 (48%) | 614 (40%) |  |
| **Race**** |  |  |  | **0.008** |
| *Non-Hispanic White* | 2,863 (75%) | 2,121 (74%) | 742 (77%) |  |
| *Non-Hispanic Black* | 1,489 (9.6%) | 1,163 (9.5%) | 326 (10.0%) |  |
| *Mexican American* | 836 (4.9%) | 723 (5.4%) | 113 (3.0%) |  |
| *Other Race - Including Multi-Racial* | 754 (6.5%) | 634 (6.5%) | 120 (6.4%) |  |
| *Other Hispanic* | 704 (4.3%) | 562 (4.4%) | 142 (3.9%) |  |
| **BMI(Kg/m²)***** |  |  |  | **<0.001** |
| *Normal(≥18.5,<25)* | 1,483 (23%) | 1,204 (24%) | 279 (20%) |  |
| *Obese(≥30)* | 2,787 (43%) | 2,063 (41%) | 724 (51%) |  |
| *Overweight(≥25,<30)* | 2,296 (34%) | 1,880 (36%) | 416 (29%) |  |
| **Marital*** |  |  |  | **0.034** |
| *Divorced* | 3,708 (62%) | 2,987 (63%) | 721 (58%) |  |
| *Married* | 2,694 (35%) | 2,033 (34%) | 661 (39%) |  |
| *Never married* | 241 (2.3%) | 180 (2.2%) | 61 (2.3%) |  |
| **Serum cotinine***** | 47 (122) | 39 (114) | 76 (142) | **<0.001** |
| **Smoking.status***** |  |  |  | **<0.001** |
| *Current* | 1,066 (14%) | 717 (12%) | 349 (23%) |  |
| *Former* | 2,192 (34%) | 1,661 (33%) | 531 (37%) |  |
| *Never* | 3,388 (52%) | 2,825 (55%) | 563 (40%) |  |
| **Education**** |  |  |  | **0.006** |
| *High educational level* | 1,601 (25%) | 1,224 (24%) | 377 (28%) |  |
| *Medium educational level* | 3,589 (63%) | 2,851 (64%) | 738 (58%) |  |
| *Primary educational level* | 1,456 (12%) | 1,128 (12%) | 328 (14%) |  |
| **Cardiovascular disease***** | 1,294 (17%) | 844 (13%) | 450 (29%) | **<0.001** |
| *Heart_failure* | 391 (4.4%) | 208 (2.8%) | 183 (10%) |  |
| *Coronary_heart_disease* | 549 (7.8%) | 350 (6.1%) | 199 (14%) |  |
| *Angina_pectoris* | 294 (4.2%) | 173 (2.8%) | 121 (9.3%) |  |
| *Heart_attack* | 534 (6.7%) | 346 (5.2%) | 188 (12%) |  |
| *Stroke* | 453 (5.3%) | 311 (4.4%) | 142 (8.6%) |  |
| **Hypertension***** |  |  |  | **<0.001** |
| *Hypertension* | 4,374 (60%) | 3,335 (58%) | 1,039 (67%) |  |
| *Non_Hypertension* | 2,272 (40%) | 1,868 (42%) | 404 (33%) |  |
| **Hyperlipidemia** |  |  |  | 0.2 |
| *Hyperlipidemia* | 5,328 (81%) | 4,147 (80%) | 1,181 (83%) |  |
| *Non_Hyperlipidemia* | 1,318 (19%) | 1,056 (20%) | 262 (17%) |  |
| **Diabetes***** |  |  |  | **<0.001** |
| *Diabetes* | 2,118 (26%) | 1,586 (24%) | 532 (31%) |  |
| *Non_Diabetes* | 4,528 (74%) | 3,617 (76%) | 911 (69%) |  |
| **CVH level***** |  |  |  | **<0.001** |
| *Low* *level (0-49)* | 1,191 (14%) | 806 (12%) | 385 (23%) |  |
| *Moderate level (50-79)* | 4,691 (70%) | 3,728 (70%) | 963 (69%) |  |
| *High level (80-100)* | 764 (16%) | 669 (18%) | 95 (8.0%) |  |
| **LE8 scores***** | 65 (14) | 66 (14) | 60 (15) | **<0.001** |
| **WBC***** | 7.19 (3.63) | 7.09 (3.92) | 7.59 (2.22) | **<0.001** |
| **NEU***** | 4.25 (1.60) | 4.17 (1.57) | 4.55 (1.68) | **<0.001** |
| **NLR***** | 2.33 (1.34) | 2.29 (1.26) | 2.46 (1.59) | **0.007** |
| **SII***** | 531 (340) | 515 (312) | 584 (422) | **<0.001** |

1Mean ± SD for continuous; n (%) for categorical

2t-test adapted to complex survey samples; chi-squared test with Rao & Scott's second-order correction

3*P < 0.05; **P < 0.01; ***P < 0.001

NHANES: National Health and Nutrition Examination Survey; CIAD: Chronic inflammatory airway diseases; CVH: Cardiovascular health; BMI: Body mass index; LE8: Life's Essential 8; WBC: White blood cell count; NEU: Neutrophil count; NLR: Neutrophil-to-Lymphocyte Ratio; SII: Systemic Immune-Inflammation Index.

# Table S4. Weighted multivariate adjusted logistic regression analysis of CIAD risk associated with different CVH levels after filling in covariate missing values

| **Regression model** | **Crude Model**  **OR (95% CI)** | **Model 1**  **OR (95% CI)** | **Model 2**  **OR (95% CI)** | **Model3**  **OR (95% CI)** |
| --- | --- | --- | --- | --- |
| **CIAD** |  |  |  |  |
| *High level (80-100)* | Reference | Reference | Reference | Reference |
| *Moderate level (50-79)* | 2.23(1.74, 2.85)*** | 2.24(1.74, 2.88)*** | 1.6(1.20, 2.14)** | 1.59(1.16, 2.16)** |
| *Low* *level (0-49)* | 4.43(3.37, 5.82) *** | 4.41(3.34, 5.83)*** | 2.22(1.49, 3.30)*** | 2.09(1.34, 3.26)** |
| *P for trend* | 0.01 | 0.01 | 8.69E-03 | 4.60E-03 |
| LE8 | 0.97(0.97, 0.98)*** | 0.97(0.97, 0.98) *** | 0.98(0.98, 0.99) *** | 0.99(0.98, 1.00) ** |
| **COPD** |  |  |  |  |
| *High level (80-100)* | Reference | Reference | Reference | Reference |
| *Moderate level (50-79)* | 3.26(1.72, 6.19)*** | 3.07(1.62, 5.82)** | 1.53(0.78, 3.00) | 1.58(0.79, 3.16) |
| *Low* *level (0-49)* | 8.5(4.25, 17.0)*** | 8.13(4.01, 16.5)*** | 2.26(1.09, 4.71)* | 2.33(1.08, 5.00)* |
| *P for trend* | 0.02 | 0.02 | 0.02 | 0.01 |
| LE8 | 0.96(0.95, 0.97) *** | 0.96(0.95, 0.97) *** | 0.99(0.98, 1.00) * | 0.99(0.98, 1.01) |
| **Asthma** |  |  |  |  |
| *High level (80-100)* | Reference | Reference | Reference | Reference |
| *Moderate level (50-79)* | 1.62(1.23, 2.14)** | 1.7(1.29, 2.25)*** | 1.41(1.00, 1.99)* | 1.44(1.03, 2.03) |
| *Low* *level (0-49)* | 2.79(2.07, 3.76)*** | 2.87(2.12, 3.88)*** | 1.97(1.17, 3.32)* | 2.04(1.23, 3.40)** |
| *P for trend* | 0.01 | 8.77E-03 | 7.93-03 | 8.94E-03 |
| LE8 | 0.98(0.97, 0.98) *** | 0.98(0.97, 0.98) *** | 0.99(0.98, 0.99) ** | 0.99(0.98, 1.00) * |
| **Chronic bronchitis** |  |  |  |  |
| *High level (80-100)* | Reference | Reference | Reference | Reference |
| *Moderate level (50-79)* | 2.59(1.47, 4.57)** | 2.59(1.45, 4.62)** | 1.62(0.89, 2.93) | 1.60(0.85, 3.01) |
| *Low* *level (0-49)* | 4.82(2.75, 8.44)*** | 4.74(2.67, 8.39)*** | 1.83(0.99, 3.38) | 1.66(0.82, 3.37) |
| *P for trend* | 4.66E-03 | 4.08E-03 | 2.29E-03 | 5.95E-04 |
| LE8 | 0.97(0.96, 0.98) *** | 0.97(0.96, 0.98) *** | 0.99(0.97, 1.00) ** | 0.99(0.97, 1.00) |

*P < 0.05; **P < 0.01; ***P < 0.001

Multiple logistic regression model: Model 1: Adjusted for baseline demographic data; Model 2: Adjusted for baseline demographic data and lifestyle factors; Model 3: Adjusted for baseline demographic data, lifestyle factors and comorbidities.

CIAD: Chronic inflammatory airway diseases; CVH: Cardiovascular health; COPD: Chronic obstructive pulmonary disease.

# Table S5. Weighted multivariable adjusted logistic regression analysis of the risk of CIAD associated with different inflammatory markers

| **Regression model** | **Crude Model**  **OR (95% CI)** | **Model 1**  **OR (95% CI)** | **Model 2**  **OR (95% CI)** | **Model3**  **OR (95% CI)** |
| --- | --- | --- | --- | --- |
| **White blood cell count** | 1.05(0.99, 1.13) | 1.06(0.98, 1.13) | 1.02(0.98, 1.06) | 1.01(0.99, 1.03) |
| **Neutrophil count** | 1.14(1.08, 1.21) * | 1.15(1.09, 1.22) *** | 1.08(1.01, 1.15) * | 1.06(1.00, 1.13) |
| **Neutrophil-to-Lymphocyte Ratio** | 1.09(1.01, 1.18) * | 1.10(1.02, 1.19) * | 1.09(1.01, 1.18) * | 1.07(0.99, 1.16) |
| **Systemic Immune-Inflammation Index_log** | 1.37(1.10, 1.69) *** | 1.37(1.10, 1.70) ** | 1.29(1.04, 1.61) * | 1.28(1.03, 1.58) * |

*P < 0.05; **P < 0.01; ***P < 0.001

Multiple logistic regression model: Model 1: Adjusted for baseline demographic data; Model 2: Adjusted for baseline demographic data and lifestyle factors; Model 3: Adjusted for baseline demographic data, lifestyle factors and comorbidities.

# Table S6. Mediating Role of Inflammatory Indicators in the Relationship between CVH Levels and CIAD

| **Mediators** | **Total effect** | |  | **Direct effect** | |  | **Indirect effect** | | |  | | **Proportion**  **Mediated** | |
| --- | --- | --- | --- | --- | --- | --- | --- | --- | --- | --- | --- | --- | --- |
|  | **Coeffcients (95% Cl)** | **P** |  | **Coeffcients (95% Cl)** | **P** |  | **Coeffcients (95% Cl)** | **P** |  | |  | |  |
| **CIAD** |  |  |  |  |  |  |  |  | |  | |  | |
| *WBC* | 4.28e-02(7.68E-03, 0.08) | 0.04 |  | -4.71E-03(-0.11, 0.08) | 0.92 |  | -4.37E-03(0.10, 0.07) | 0.92 | |  | | 0.92 | |
| *NEU* | 0.04(5.06E-03, 0.08) | 0.04 |  | 0.04(2.02E-03, 0.08) | 0.05 |  | 2.67E-03(5.97E-04, 1E-04) | <2E-16 | |  | | 0.04 | |
| *NLR* | 0.04(8.67E-03, 0.07) | 0.02 |  | 0.02(-0.03, 0.12) | 0.32 |  | 0.02(-0.02, 0.11) | 0.32 | |  | | 0.32 | |
| *SII* | 0.04(7.05E-03, 0.08) | 0.04 |  | 0.08(2.17E-03, 0.25) | 0.05 |  | 0.08(2.28E-03, 0.25) | 0.05 | |  | | 0.05 | |
| **COPD** |  |  |  |  |  |  |  |  | |  | |  | |
| *WBC* | 0.02(3.3E-04, 0.04) | 0.05 |  | 5.13E-03(-0.07, 0.11) | 0.66 |  | 3.45E-03(-0.60, 0.11) | 0.66 | |  | | 0.66 | |
| *NEU* | 0.02(-2.91E-03, 0.04) | 0.08 |  | 0.02(4.93E-03, 0.04) | 0.1 |  | 0.02(4.81-03, 0.04) | 0.1 | |  | | 0.08 | |
| *NLR* | 0.02(4.71E-02, 0.04) | 0.02 |  | 0.03(-0.05, 0.12) | 0.24 |  | 0.02(-0.04, 0.11) | 0.24 | |  | | 0.24 | |
| *SII* | 0.02(-4.35E-03, 0.04) | 0.1 |  | 0.06(-0.56, 0.35) | 0.1 |  | 0.05(-0.62, 0.33) | 0.1 | |  | | 0.1 | |
| **Chronic bronchitis** |  |  |  |  |  |  |  |  | |  | |  | |
| *WBC* | 0.02(-3.53E-03, 0.04) | 0.16 |  | -1.95E-03(-0.18, 0.29) | 0.96 |  | -1.40E-03(-0.18, 0.29) | 0.96 | |  | | 0.96 | |
| *NEU* | 0.02(-3.17E-03, 0.04) | 0.16 |  | 0.05(-0.39,0.23) | 0.18 |  | 0.04(-0.40,0.22) | 0.18 | |  | | 0.18 | |
| *NLR* | 0.02(-2.86E-03, 0.04) | 0.1 |  | 0.02(-0.03, 0.25) | 0.30 |  | 0.01(-0.02, 0.25) | 0.30 | |  | | 0.30 | |
| *SII* | 0.02(-7.66E-05, 0.04) | 0.05 |  | 0.06(-0.04, 0.35) | 0.06 |  | 0.05(-0.04, 0.34) | 0.06 | |  | | 0.06 | |
| **Asthma** |  |  |  |  |  |  |  |  | |  | |  | |
| *WBC* | 0.03(-7.16E-04, 0.05) | 0.08 |  | 1.40E-03 (-0.18, 0.14) | 0.96 |  | 1.16E-03 (-0.17, 0.13) | 0.96 | |  | | 0.96 | |
| *NEU* | 0.03(-4.95E-03, 0.06) | 0.14 |  | 0.07(-0.53, 0.44) | 0.14 |  | 0.06(-0.54, 0.43) | 0.14 | |  | | 0.14 | |
| *NLR* | 0.02(-6.23E-03, 0.05) | 0.16 |  | 0.02(-0.18, 0.12) | 0.3 |  | 0.02(-0.19, 0.12) | 0.3 | |  | | 0.3 | |
| *SII* | 0.02(-0.01, 0.05) | 0.16 |  | 0.06(-0.26, 0.81) | 0.18 |  | 0.05(-0.27, 0.81) | 0.18 | |  | | 0.18 | |

CIAD: Chronic inflammatory airway diseases; CVH: Cardiovascular health; COPD: Chronic obstructive pulmonary disease; WBC: White blood cell count; NEU: Neutrophil count; NLR: Neutrophil-to-Lymphocyte Ratio; SII: Systemic Immune-Inflammation Index.

# Table S7. Characteristics of participants by age (NHANES 2013-2018)

| **Characteristic** | **Overall**, N = 11258 (100%)^1,2^ | **<50**, N = 5522 (52%)^1,2^ | **≧50**, N = 5736 (48%)^1,2^ | **P Value**^3^ |
| --- | --- | --- | --- | --- |
| **Sex** |  |  |  | 0.061 |
| *Female* | 5,990 (53%) | 3,005 (52%) | 2,985 (54%) |  |
| *Male* | 5,268 (47%) | 2,517 (48%) | 2,751 (46%) |  |
| **Race***** |  |  |  | **<0.001** |
| *Non-Hispanic White* | 4,425 (67%) | 1,952 (59%) | 2,473 (75%) |  |
| *Non-Hispanic Black* | 2,437 (11%) | 1,176 (12%) | 1,261 (9.3%) |  |
| *Other Race - Including Multi-Racial* | 1,635 (8.3%) | 983 (10.0%) | 652 (6.5%) |  |
| *Mexican American* | 1,595 (8.2%) | 861 (11%) | 734 (4.9%) |  |
| *Other Hispanic* | 1,166 (6.1%) | 550 (7.6%) | 616 (4.4%) |  |
| **BMI***** |  |  |  | **<0.001** |
| *Normal(≥18.5,<25)* | 2,924 (27%) | 1,627 (30%) | 1,297 (23%) |  |
| *Obese(≥30)* | 4,693 (41%) | 2,256 (39%) | 2,437 (43%) |  |
| *Overweight(≥25,<30)* | 3,641 (32%) | 1,639 (31%) | 2,002 (34%) |  |
| **Marital***** |  |  |  | **<0.001** |
| *Divorced* | 5,884 (56%) | 2,631 (50%) | 3,253 (63%) |  |
| *Married* | 5,011 (41%) | 2,723 (47%) | 2,288 (35%) |  |
| *Never married* | 363 (2.3%) | 168 (2.6%) | 195 (2.1%) |  |
| **Serum cotinine**** | 51.9 (124.3) | 56.9 (126.1) | 46.3 (122.0) | **0.005** |
| **Smoking status***** |  |  |  | **<0.001** |
| *Current* | 2,029 (17%) | 1,144 (20%) | 885 (14%) |  |
| *Former* | 2,714 (25%) | 826 (18%) | 1,888 (34%) |  |
| *Never* | 6,515 (58%) | 3,552 (63%) | 2,963 (52%) |  |
| **Education** |  |  |  | 0.10 |
| *9-11th Grade (Includes 12th grade with no diploma)* | 1,257 (8.1%) | 610 (8.2%) | 647 (8.0%) |  |
| *College Graduate or above* | 2,977 (33%) | 1,577 (34%) | 1,400 (32%) |  |
| *High School Grad/GED or Equivalent* | 2,577 (23%) | 1,205 (22%) | 1,372 (24%) |  |
| *Less Than 9th Grade* | 842 (3.7%) | 262 (3.2%) | 580 (4.2%) |  |
| *Some College or AA degree* | 3,605 (32%) | 1,868 (33%) | 1,737 (32%) |  |
| **CVD***** |  |  |  | **<0.001** |
| *CVD* | 1,248 (8.8%) | 140 (1.9%) | 1,108 (16%) |  |
| *Non-CVD* | 10,010 (91%) | 5,382 (98%) | 4,628 (84%) |  |
| **Hypertension***** |  |  |  | **<0.001** |
| *Hypertension* | 4,969 (39%) | 1,219 (20%) | 3,750 (59%) |  |
| *Non-Hypertension* | 6,289 (61%) | 4,303 (80%) | 1,986 (41%) |  |
| **Hyperlipidemia***** |  |  |  | **<0.001** |
| *Hyperlipidemia* | 7,914 (69%) | 3,209 (57%) | 4,705 (82%) |  |
| *Non-Hyperlipidemia* | 3,344 (31%) | 2,313 (43%) | 1,031 (18%) |  |
| **Diabetes***** |  |  |  | **<0.001** |
| *Diabetes* | 2,335 (16%) | 456 (6.8%) | 1,879 (26%) |  |
| *Non-Diabetes* | 8,923 (84%) | 5,066 (93%) | 3,857 (74%) |  |
| **LE8 Score***** | 69 (15) | 72 (14) | 65 (14) | **<0.001** |
| **CVH level***** |  |  |  | **<0.001** |
| *Low* | 1,428 (10%) | 416 (6.5%) | 1,012 (14%) |  |
| *Moderate* | 7,474 (65%) | 3,426 (61%) | 4,048 (69%) |  |
| *High* | 2,356 (25%) | 1,680 (33%) | 676 (17%) |  |
| **LE8***** | 69 (15) | 72 (14) | 65 (14) | **<0.001** |
| **WBC***** | 7.41 (3.14) | 7.57 (2.35) | 7.22 (3.82) | **<0.001** |
| **NEU***** | 4.36 (1.74) | 4.46 (1.86) | 4.26 (1.60) | **<0.001** |
| **NLR***** | 2.18 (1.12) | 2.07 (0.97) | 2.31 (1.25) | **<0.001** |
| **SII** | 520 (303) | 515 (290) | 526 (316) | 0.2 |
| **CIAD*** |  |  |  | **0.016** |
| *CIAD* | 2,262 (20%) | 1,018 (19%) | 1,244 (22%) |  |
| *Non-CIAD* | 8,996 (80%) | 4,504 (81%) | 4,492 (78%) |  |
| **COPD***** | 445 (3.6%) | 49 (0.7%) | 396 (6.8%) | **<0.001** |
| **Chronic bronchitis***** | 686 (6.0%) | 240 (4.6%) | 446 (7.6%) | **<0.001** |
| **Asthma*** | 1,730 (15%) | 887 (16%) | 843 (14%) | **0.043** |

1Mean ± SD for continuous; n (%) for categorical

2t-test adapted to complex survey samples; chi-squared test with Rao & Scott's second-order correction

3*P < 0.05; **P < 0.01; ***P < 0.001

NHANES: National Health and Nutrition Examination Survey; CIAD: Chronic inflammatory airway diseases; CVH: Cardiovascular health; BMI: Body mass index; LE8: Life's Essential 8; WBC: White blood cell count; NEU: Neutrophil count; NLR: Neutrophil-to-Lymphocyte Ratio; SII: Systemic Immune-Inflammation Index.

# Figure S2. Trend line chart of CIAD and its subtypes with age cutoff at 50 years


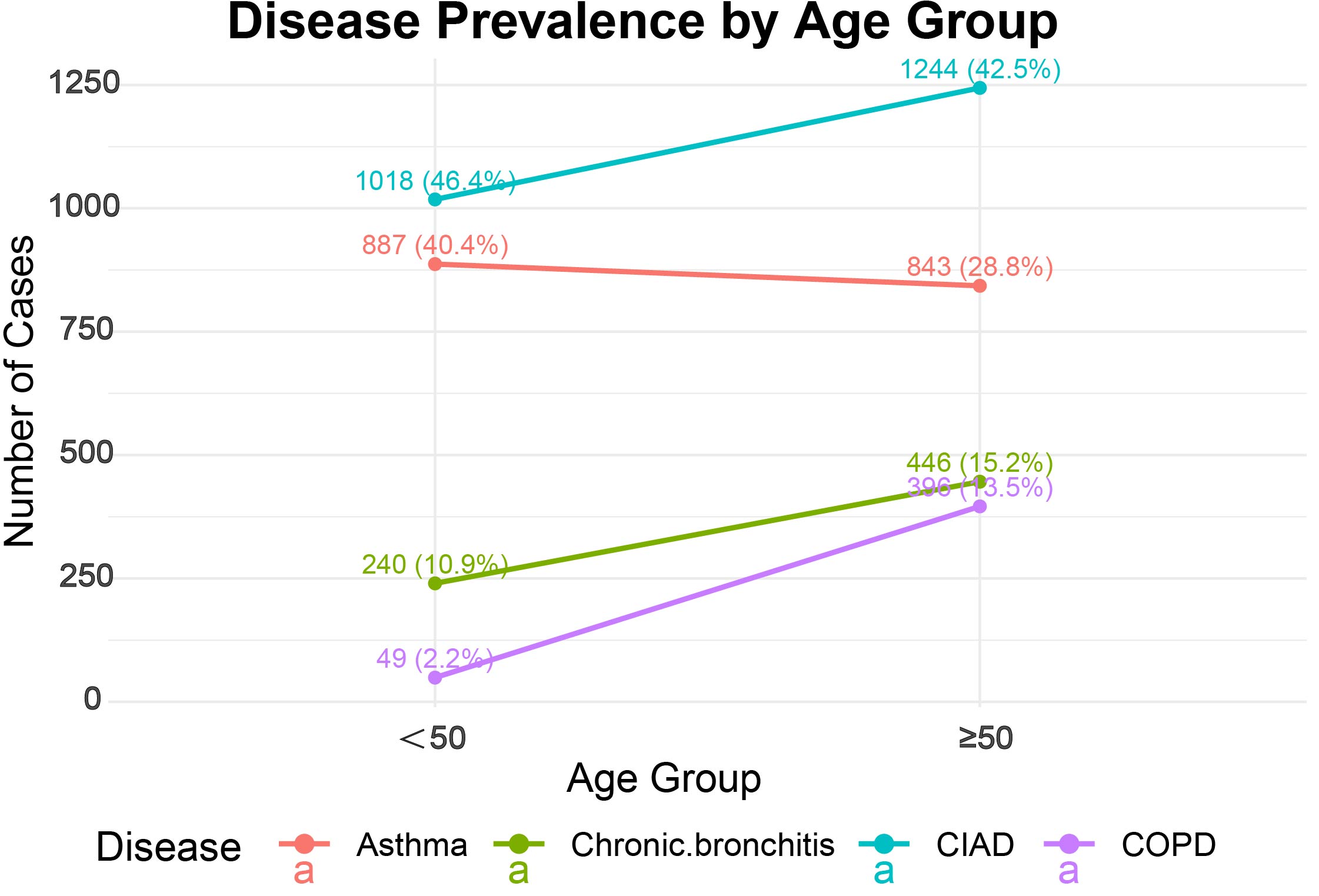


This figure compares the prevalence of CIAD and its subtypes in populations under and over 50 years of age. In the color definitions, orange-red represents asthma, green represents chronic bronchitis, purple represents COPD, and blue represents CIAD. The percentages in parentheses indicate the proportion of the disease within the corresponding age group (e.g., asthma accounts for 2.2% in the <50 years group). The results show that the number of CIAD cases in the under 50 group is 1,018 (46.4%), while in the ≥50 group, it is 1,244 (42.5%). Additionally, the proportion of COPD in the >50 group begins to increase significantly, while the number of asthma cases not only decreases in quantity but also shows a sharp decline in proportion.

# Figure S3. Trend line chart of CIAD and its subtypes across different age groups


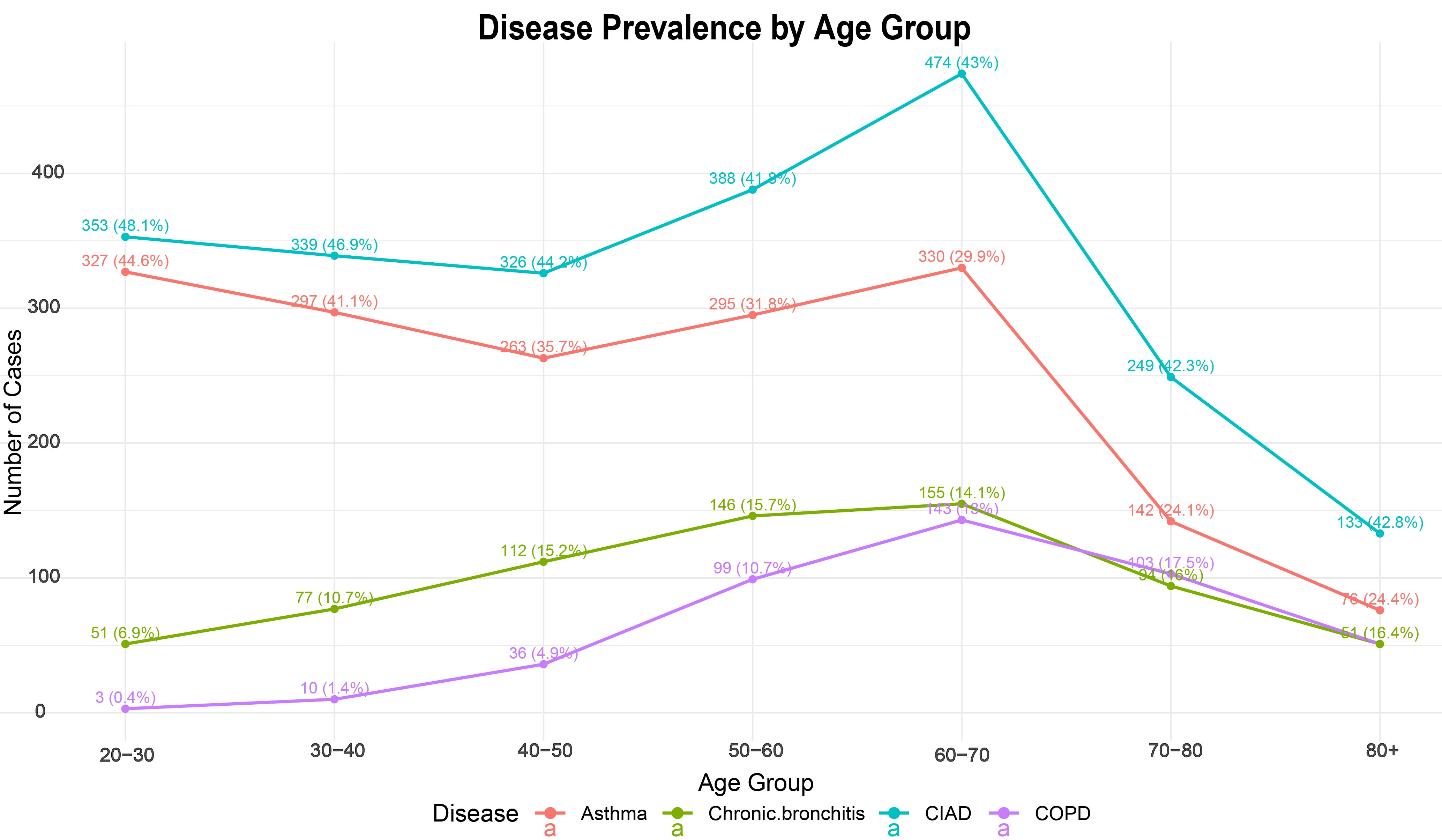


This figure displays the distribution of disease prevalence across seven different age groups. The number of cases fluctuates with increasing age. In the color definitions, orange-red represents asthma, green represents chronic bronchitis, purple represents COPD, and blue represents CIAD. The percentages in parentheses indicate the proportion of cases for that disease within the corresponding age group. Overall, it can be observed that the proportion of chronic bronchitis and COPD is continuously rising in the older age groups, while asthma has a higher proportion in the younger groups, which declines as age increases.
